# Encoding of naturalistic optic flow by motion sensitive neurons of nucleus rotundus in the zebra finch (*Taeniopygia guttata*)

**DOI:** 10.3389/fnint.2013.00068

**Published:** 2013-09-20

**Authors:** Dennis Eckmeier, Roland Kern, Martin Egelhaaf, Hans-Joachim Bischof

**Affiliations:** ^1^Neuroethology Group, Department of Behavioural Biology, Bielefeld UniversityBielefeld, Germany; ^2^Department of Neurobiology, Bielefeld UnviersityBielefeld, Germany; ^3^The Center of Excellence Cognitive Interaction Technology (CITEC), Bielefeld UniversityBielefeld, Germany

**Keywords:** bird, zebra finch, vision, nucleus rotundus, tectofugal visual system, optic flow, self-motion, depth perception

## Abstract

The retinal image changes that occur during locomotion, the optic flow, carry information about self-motion and the three-dimensional structure of the environment. Especially fast moving animals with only little binocular vision depend on these depth cues for maneuvering. They actively control their gaze to facilitate perception of depth based on cues in the optic flow. In the visual system of birds, nucleus rotundus neurons were originally found to respond to object motion but not to background motion. However, when background and object were both moving, responses increased the more the direction and velocity of object and background motion on the retina differed. These properties may play a role in representing depth cues in the optic flow. We therefore investigated, how neurons in nucleus rotundus respond to optic flow that contains depth cues. We presented simplified and naturalistic optic flow on a panoramic LED display while recording from single neurons in nucleus rotundus of anaesthetized zebra finches. Unlike most studies on motion vision in birds, our stimuli included depth information. We found extensive responses of motion selective neurons in nucleus rotundus to optic flow stimuli. Simplified stimuli revealed preferences for optic flow reflecting translational or rotational self-motion. Naturalistic optic flow stimuli elicited complex response modulations, but the presence of objects was signaled by only few neurons. The neurons that did respond to objects in the optic flow, however, show interesting properties.

## Introduction

Optic flow is a reliable source of depth information for animals (Gibson, 1950; Koenderink, 1986; Simpson, 1993; Cornilleau-Pérès and Gielen, 1996) and is successfully used in machine based depth reconstruction (e.g., Yang et al., 2012). During translational self-motion the images of objects in different distances move on the retina with different velocities (Koenderink, 1986). The relative motion of images thus allows the perception of the three dimensional structure of the environment. Stereo vision is probably the best known mechanism for depth perception. However, it demands a large binocular visual field and the range in which it is applicable depends on the distance between the eyes. For fast moving animals with only short ranged stereo vision, such as flying insects and birds, optic flow is the main source for depth information (Egelhaaf et al., 2012). Zebra finches have laterally positioned eyes that are very close to each other. Stereo vision cannot provide depth information in a sufficient distance range when the birds navigate in clustered environments like tree tops or bushes. They most likely rely on optic flow based depth cues. Accordingly birds were previously shown to use optic flow for orientation during flight (Bhagavatula et al., 2011).

To optimize depth perception from optic flow an animal needs to avoid image motion from rotational gaze shift. Depth cues in image motion are generated by the translational component of self-motion (Koenderink, 1986; Kern et al., 2006). During a translational gaze shift, for instance during a straight flight, the images of objects move across the birds' retinae on trajectories which emanate from a common “point of expansion” that coincides with the heading direction. The retinal images of the objects enlarge and move toward the lateral visual field. When the animal passed the objects, their images converge into another common point at the back (“point of contraction”). Therefore, the images of objects moving toward the animal expand while those moving away in the back contract; images of objects that are far away move slower than those of near objects. The changes in size and velocity can be used to estimate distances to and among objects (Gibson, 1950; Koenderink, 1986). Such differential motion of images on the retina is referred to as “motion parallax.” The term “motion parallax” is, however, also often used specifically for the case when an observer fixates an object during translational locomotion. In this special case the retinal images of objects in front and behind the fixated depth plane move in opposite directions. In humans, motion parallax during fixation is discussed as an important depth cue even when the observer is not moving voluntarily (Aytekin and Rucci, 2012). While translational self-motion generates depth cues in the optic flow, pure rotational gaze shifts do not produce depth information because the angular velocities of all objects are the same independent of the distance, and there is also no change in image size (Koenderink, 1986).

Zebra finches maximize the time during which they can perceive depth from optic flow by reducing rotational gaze shift to short time intervals (Eckmeier et al., 2008). While the birds perform a smooth curve around an obstacle, they keep the orientation of their heads constant relative to the environment. They only change head orientation in fast, “saccadic” head turns (Eckmeier et al., 2008). Using an analogous strategy, blowflies and honey bees reduce the rotational gaze shifts by flying in straight lines and only changing flight direction in fast body turns (Schilstra and van Hateren, 1998; Boeddeker et al., 2010).

In order to estimate the distance to a certain static object in the environment the brain needs to process the relative differences in motion velocity of images that constitute the optic flow. Self-motion induces optic flow throughout the visual field, whereas object motion appears only in isolated regions of the visual field. In birds, such whole field motion is mainly processed by the accessory optic system and the pretectal nucleus lentiformis mesencephali (Simpson et al., 1988; Frost et al., 1990; Pakan and Wylie, 2006; Wylie, 2013). However, the accessory optic system appears mainly to involve the processing of self-motion cues that are used, for instance for gaze stabilization (Wylie, 2013).

The tectofugal visual system, on the other hand, is implicated in processing properties of single objects, including object motion (review: Bischof and Watanabe, 1997). So called “2d” neurons code for the movement of small objects across the retina (Frost et al., 1990) while “3d” neurons respond to stimuli moving toward the eye of the bird (“looming” stimuli; optic tectum: Wu et al., 2005, nucleus rotundus: Wang and Frost, 1992, entopallium: Xiao et al., 2006). The responses of neurons in the tectofugal visual system to object motion were modulated by background motion while no response was found when the motion of the object and the background were identical (nucleus rotundus: Frost et al., 1990; entopallium: Xiao and Frost, 2009).

Nucleus rotundus in the tectofugal visual system therefore is a good candidate area for the processing of depth cues in the optic flow through relative object motion. To investigate how motion sensitive neurons in nucleus rotundus react to the visual input experienced during flight, we presented optic flow stimuli coherent with self-motion in a three dimensional environment on a panoramic LED display covering almost the whole visual field of the birds (Lindemann et al., 2003). The stimuli included a naturalistic reconstruction of what an unrestrained zebra finch had seen while flying around an obstacle (Eckmeier et al., 2008). Artificial stimuli represented optic flow from pure translational and rotational self-motion in addition to simple “looming” objects. The population of cells we recorded contained a large variance in the response to our stimuli between single neurons.

## Materials and methods

All experimental procedures were performed in accordance with the German Law on the Protection of Animals and were approved by the local government, Landesamt für Natur, Umwelt und Verbraucherschutz Nordrhein-Westfalen, approval number AZ 9.93.2.10.36.07.105.

### Animals and preparation

Seventeen zebra finches (*Taeniopygia guttata*) from the department's stock were examined. Birds were anaesthetized by an injection of urethane (SIGMA Diagnostics, 0.01 ml, 20% PBS) into the flight muscle. When reflexes were not observed any longer, the bird was fixed by its head using a stereotaxic head holder (Bischof, 1981). Xylocain Gel (2%, Astra Zeneca GmbH, Wedel, Germany) was applied to the skin of the ear holes for additional local anesthesia.

**Figure 1:**
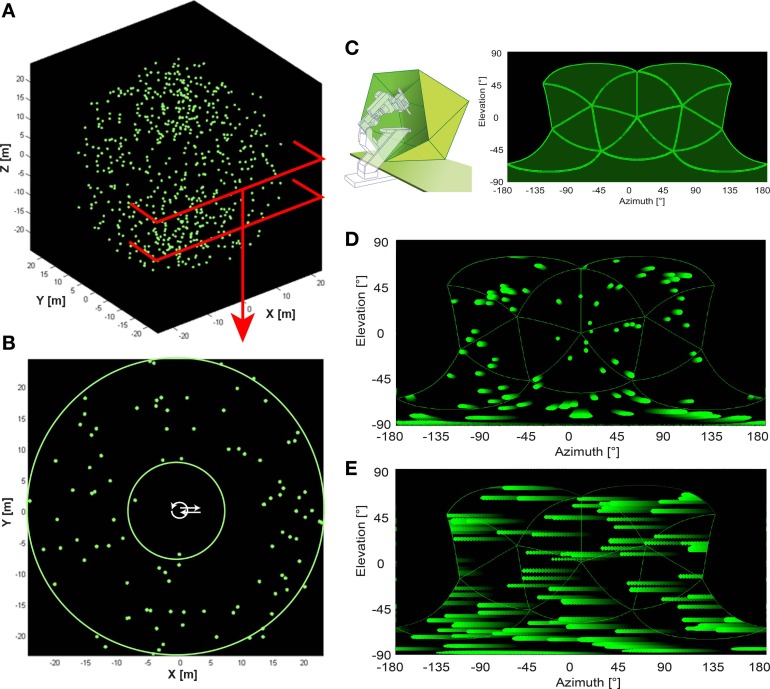
**Artificial optic flow stimuli. (A)** The virtual environment used for artificial optic flow stimuli is a “starfield” of 640 pseudo-randomly distributed spheres (green; 0.3 m radius) in a dark background. **(B)** shows a section of the starfield that includes the origin. Spheres were positioned only at distances between the two green circles (between 7.5 and 25 m distance from origin in all directions) leaving the center free of objects. Trajectories were positioned within the free central area (white arrows). **(C)** Left panel shows a schematic of the panoramic LED display, “FliMax.” FliMax consists of 14 triangular circuit boards each equipped with 512 green LEDs. The electrophysiology stand including micromanipulator and electrode holder is indicated by the dotted contour. The bird was placed on the plate in the center of FliMax. **(C)** Right panel indicates the parts of the visual field covered by FliMax as a plot of spherical coordinates: 240° (−120 to +120°) azimuth at 0° elevation and from 60 to −90° elevation of the visual field. **(D,E)** show the object motion within the boundaries of FliMax for translational and rotational gaze shift, respectively. The plot is in spherical coordinates. Object color changes from dark green to light green with time. Note that in the translational condition objects move in different directions and velocities in dependence of their position in space. In the rotational stimulus there is no difference in object motion.

**Figure 2:**
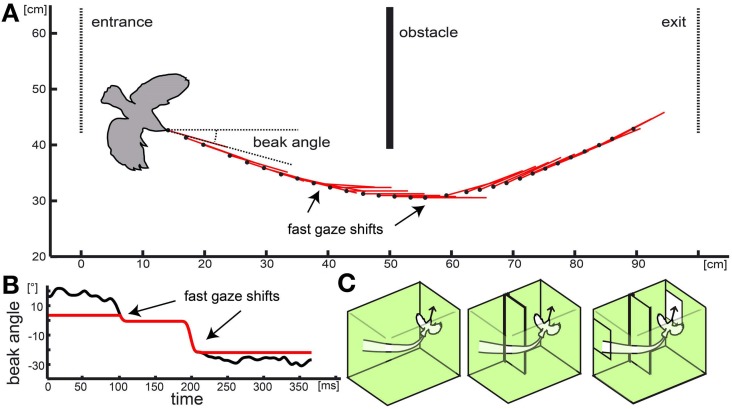
**Naturalistic stimulus: real flight trajectory and head orientations were used to reconstruct optic flow in three different virtual environments.** In **(A)** the black dots show the position of the basis of the beak for every 6th frame of the movie (every 12th ms). Red lines indicate head orientation. Obstacle, entrance, and exit are depicted as solid or dotted lines, respectively. The bird contour is approximately correct relative size. **(B)** shows the progression of the beak orientation angle (relative to the environment) over time used for the naturalistic stimulus. Black line shows beak orientation as measured including residual yaw rotations. Red line shows progression of the head orientation without the rotational fluctuations to test the influence of purely translational and rotational head movement on response properties. In **(C)** the different cage models are shown. One consisted of the empty cage (left schema) with no obstacle or window, in the second (center schema) the obstacle was added, and in the third condition (right schema) obstacle and windows were included.

Feathers were removed and the skin was incised and retracted to expose the skull at the desired positions for electrode placement. The skull was then opened by removing the two bone layers. The dura was kept intact until it was penetrated by the electrode. Both eyelids were fixed by surgical adhesive in an open position shortly before the experiment started. The nictitating membrane was kept intact to protect the eye from desiccation during the experiment. During anesthesia the nictitating membrane remains open and blinks in response to careful touch stimulation. The head holder mentioned above was attached to a plate on which the bird's body was placed. The two pieces were then mounted on a stand which carried the micro-manipulator, the pre-amplifier, and electrodes positioned in the correct angle for a stereotaxic approach (Figures 1, 2).

### Extracellular single cell electrophysiology and data acquisition

The reference electrode was clamped to the skin of the head and moistened with saline (0.9%). The recording electrode (tungsten in glass micro-electrode TM31A10, World Precision Instruments, Inc., Sarasota, USA, 0.9–1.0 MΩ, tip diameter 1–2 μm, 1 μm insulation) was positioned according to coordinates taken from the stereotaxic atlas of the zebra finch (Nixdorf-Bergweiler and Bischof, 2007). After penetration of the dura, the electrode tip was advanced to a depth of 500 μm. Then a grounded hood of fine wire mesh was attached to the stand to shield bird and electrode from electromagnetic noise caused by the stimulus apparatus. The hood did not obscure the bird's view. The stand was then placed within the spherical stimulus device (FliMax, see below), the head positioned at the center of the apparatus.

The electrode was advanced slowly in steps of 2 μm by a motorized microdrive. We began the search for motion sensitive neurons approximately 500 μm above the target area. Visual stimuli were presented to the bird using a flashlight while further advancing the electrode. The amplified neuronal responses were visualized by an oscilloscope and also monitored acoustically by a loudspeaker. When a neuron was responding to the moving flashlight, its responses were tested as described below. After reaching a depth of 5500 μm (~500 μm below the nucleus rotundus according to Nixdorf-Bergweiler and Bischof, 2007), the electrode was retracted completely and re-inserted at a slightly different coordinate for a new approach. The distance between recording sites and between penetration sites was at least 50 μm to avoid recording the same neuron twice.

The received signal was amplified (× 1000) and band pass filtered (300 Hz lower, 20 kHz upper cut-off frequency; A-M Systems Model 1800) before it was digitized (CED 1401 mkII, Cambridge Electronic Design) and stored (Spike 2 recording software, Cambridge Electronic Design). The activity of single neurons within a recording was separated offline using the spike sorting function provided by Spike 2 (a template matching procedure).

We marked the location of the electrode at the end of an experiment with an electrical lesion. The brain was removed and stored for at least 2 days in fixative (4% paraformaldehyde in phosphate buffered saline (PFA) followed by 30% saccharose in PFA). Coronal 40 μm sections were cut, mounted on glass slides, and stained with Giemsa dye (SIGMA Diagnostics, St. Louis, USA).

### Stimulus presentation device FliMax

The stimulation device FliMax (Lindemann et al., 2003) was initially constructed for experiments with flies. It is a segment of an icosahedron (44.8 cm in diameter; Figure 1C). More than 7000 green light emitting diodes (diameter 5 mm, wavelength 567 nm; WU-2-53GD, Vossloh Wustlich Opto, Germany) are positioned equally spaced on 14 triangular circuit boards. The illumination of these diodes can be controlled by a computer program, so contrast can be changed if necessary. In principle, the device can be seen as a spherical computer screen with low spatial (LED separation 2.3°) but high temporal resolution (370 fps). The maximum luminance averaged over the array of LEDs is 420 cd/m^2^. On this LED screen optic flow stimuli could be presented that were previously computed as described below.

FliMax covers most but not all of the visual field of a zebra finch (Figure 1C). It illuminates an area of 240° (−120 to +120°) azimuth at 0° elevation and from +60 to −90° elevation (the origin being the center of the display). The visual field of the zebra finch covers an area of ~300° (−150 to + 150°) azimuth (Bischof, 1988). According to Martin (2007) the eye size of the zebra finch (~4.2 mm diameter) indicates that there may be no blind area above the head. The outmost areas of the rear and top visual field could thus not be stimulated by the device. The literature does not describe visual acuity and spatial resolution of the zebra finch retina. The visual resolution of the zebra finches is probably much higher than the resolution of FliMax. We can assume, however, that this does not much affect optic flow processing and perception by the bird. Even for human observers the percept of optic flow as generated in FliMax was stunning, even though no spatial details can be resolved. Finally, when the movies for FliMax are generated, spatial smoothing is included to limit spatial aliasing.

#### Motion stimuli

To produce a stimulus movie, a virtual three dimensional environment had to be designed. Within this virtual environment a trajectory was defined that represented the motion of the bird's head. We then calculated a movie from the bird's perspective following the trajectory within the virtual environment.

*Looming stimuli* were presented at different angular positions in the visual field. The stimulus resembled a bright disc (diameter 60 cm) at a distance of 3.5 m (angular disc size 9.73°) which then approached with a speed of 3.5 m/s resulting in a stimulus duration of 1 s. The stimuli differed by the angular position of the expanding disc in the visual field. We chose five different angular positions: frontal (0° elevation and azimuth), above frontal (+45° elevation; 0° azimuth), below frontal (−45° elevation, 0° azimuth), fronto-lateral right (+45° azimuth, 0° elevation) and fronto-lateral left (−45°azimuth, 0° elevation).

*Simplified optic flow stimuli* were characterized by optic flow consistent with translational (forward/backward) and rotational (clockwise/counter clockwise) self-motion within an artificial environment (Figure 1). This artificial environment was a virtual three dimensional ‘star field’ consisting of 640 bright globes (60 cm radius), distributed pseudo-randomly in the space around the starting point of the virtual self-motion (Figure 1A). The background was dark. The nearest distance of one of the globes to this starting point was 7.5 m (sphere size 4.6°); the maximum was 25 m (Figure 1B) (sphere size 1.4°). Spheres further away than ~15 m from the observer were not presented on FliMax because they were too small (but, of course, appeared when approached). A stimulus consisted of two optic flow phases moving in opposite directions, each preceded by 1 s of still image. The simulated velocity of the moving bird was always 3.5 m/s (translational stimuli) or 400°/s (rotational stimuli). The rotational velocity of 400°/s was chosen to be at the upper limit for compensatory head movements. This was estimated in an earlier study on the optocollic reflex in zebra finches (Eckmeier and Bischof, 2008). The translational velocity of 3.5 m/s was chosen to be at the upper limit of flight speed measured during our free flight experiments (Eckmeier et al., 2008). We therefore tested the visual system under conditions similar to fast locomotion.

While the angular retinal image velocity for all objects within the rotational stimuli was identical, this was not the case with the translation velocity. The velocities and the size of the object images on the retina vary dependent on the distance of the objects to the animal and on the position relative to the point of expansion (Figures 1D,E). The image velocity for most objects in the simplified self-motion varied between 0 and 20°/s, however, extremes reached up to 105°/s vertical motion and 865°/s horizontal motion, respectively.

*Naturalistic optic flow stimuli (Figure 2; supplementary video1)* were generated by following a flight trajectory that was measured during our previous study (Eckmeier et al., 2008) within a virtual reconstruction of the flight arena the flight was observed in. The original test cage consisted of a central flight arena of 1 m width and two outer compartments. The walls of the central flight arena were lined with textured paper. The birds entered the flight arena through a window from one outer compartment and left it through an exit window into the second outer compartment at the opposite side. Two high-speed cameras above and in front of the cage recorded the flight. To advance to the exit window, the test animals had to navigate around an obstacle (incomplete wall) at the middle of the flight arena (Figure 2).

We manipulated the stimulus by altering the virtual environment as well as the trajectory. We aimed to compare the neuronal response to the optic flow experienced during a flight within an “empty cage” and within a “complete cage” including the obstacle and windows. In addition we tested a “cage with obstacle” which included the obstacle but not the windows (Figure 2C). In a fourth stimulus, we eliminated residual head rotations from the inter-saccadic intervals (Figure 2B).

#### Analysis

Identified action potentials were summed across trials, binned (2 ms bin size), and translated into firing rates (spikes/s). The resulting peristimulus-time histograms were further analyzed with custom made Matlab® (Mathworks) scripts.

In general, firing rates in motion sensitive neurons during still image presentation were low and used as baseline activation. Activation by motion was obvious in comparison. Accordingly we could set a low threshold (average baseline activity plus two times the standard deviation of the baseline activation) to determine whether a neuron responded. Response latencies were measured as the time delay between stimulus onset and earliest exceedance of this threshold.

To quantify response preferences between rotational and translational neurons, we compared the mean baseline subtracted tonic activation during motion stimulation. We averaged the response during the last 500 ms of each stimulus period for translation (*R*_*t*_) and rotation (*R*_*r*_) stimuli, excluding phasic response effects to the stimulus onset. The score was calculated by dividing the difference between these values by their sum: (*R*_*r*_ − *R*_*t*_)/(*R*_*r*_ + *R*_*t*_). The score ranges from −1 (response to translational motion only) to 1 (response to rotational motion only). For description purposes, the overall score range was subdivided in three ranges of equal size (0.66). In the range of −1 to −0.33 neurons fired at least twice as often during translational stimuli than during rotational stimuli, in the range 0.33–1 they fired at least twice as often during rotational stimuli than during translational stimuli. This subdivision was used to describe preferences of the neurons and is not intended to indicate a clustering of cells according to their response properties.

For looming responses we calculated for each cell a mean response direction. We summed vectors pointing from the center into the direction of the object for each respective stimulus. The length of these contributing vectors corresponded to the maximum firing rate measured during stimulus presentation and was normalized to the sum of all five maximum firing rates.

Principle component analyses (PCA) and hierarchical clustering of all responses to the different stimuli were performed with Matlab. The PCA we performed on the responses to artificial stimuli. The hierarchical clustering was performed on averaged responses of single cells as well as single responses for naturalistic stimuli and was used to sort the responses by similarity.

For a first analysis of naturalistic stimuli we determined motion parameters of the optic flow within the receptive field of the recorded neuron. To assess the image velocities the neuron was responding to, we used averaged local velocities within the receptive field for each frame of the stimulus using Matlab® (custom toolbox developed at the Department of Neurobiology, Bielefeld University, Germany). We also determined the average distance to objects that appeared in the receptive field of each neuron. The resulting time courses of vertical and horizontal velocities as well as distances were compared to the time courses of the neuronal responses.

We estimated whether the response to naturalistic stimuli could be predicted by the response to artificial stimuli. We sorted the response to naturalistic stimuli by parameters describing the response to artificial stimuli that appeared to discriminate cells well: rotational preference and response latency to looming stimuli. We then visually examined whether similar response patterns to naturalistic stimuli would appear close to each other.

#### Receptive field estimation

To be able to estimate local effects of optic flow in the naturalistic stimulus situation (see above) the position of the receptive field within the visual space and a rough estimation of its extension were necessary. We did not intend to repeat for the zebra finch the detailed measurement of size and shape of receptive fields within nucleus rotundus as it was previously done (Wang et al., 1993; Schmidt and Bischof, 2001). Due to time constraints we developed a quick method to estimate the size and position of the receptive field. A vertically oriented stripe (a semi-circle in FliMax) was horizontally rotated around the bird's head (around yaw axis), and a horizontally oriented semi-circle was rotated around the bird's head vertically (around pitch axis) at 100°/s. The semi-circle rotated by 360° in one direction and then by 360° in the opposite direction for both scans. When the stimulus semi-circle entered the receptive field, a transient response was measured. We then combined the responses to vertical and horizontal scans to a map spanning −90 to +90° elevation and −180 to +180° azimuth in steps of 5°. Response maxima marked the corners of a rectangle bordering the receptive field.

## Results

**Figure 3:**
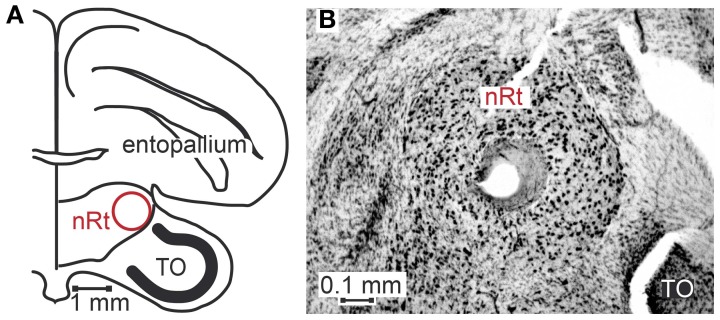
**Location of nucleus rotundus and histological verification of recording sites.** Panel **(A)** shows a schematic of a coronal section of the zebra finch brain according to Nixdorf-Bergweiler and Bischof (2007), 2.4 cm anterior to the Y-point (origin of coordinate system). **(B)** shows a photograph of a Giemsa-stained section. In the center of nucleus rotundus (nRt) a lesion is visible. In the lower right corner of the photograph a small part of the optic tectum (TO) can be seen.

Seventy-six motion sensitive neurons were recorded throughout nucleus rotundus of 17 zebra finches during visual stimulation. All simplified stimuli described in the methods section were presented to every neuron 30–35 times. We further presented 1, 2 or all four of the naturalistic stimuli 30–35 times. Sixty-four neurons were recorded from the right, twelve from the left hemisphere. Figure 3A is a schematic drawing of a coronal section of the zebra finch brain depicting nucleus rotundus. According to the stereotaxic atlas of the zebra finch (Nixdorf-Bergweiler and Bischof, 2007) its center is located at 2.6 mm anterior, 2 mm lateral, and about 4.9 mm deep from the “Y”-point, the origin of the coordinate system. This center is marked by a lesion in Figure 3B, a Nissl stained coronal section including nucleus rotundus and adjacent tissue. Nucleus rotundus can be clearly distinguished from the surrounding by its large, darkly stained neurons. It is an almost spherical structure with a diameter of about 0.9 mm.

Please note that we refer to optic flow stimuli consistent with translational gaze shift as “translational” and optic flow stimuli consistent with rotational gaze shift as “rotational.”

### Artificial optic flow stimuli reveal a distribution of preferences between optic flow from rotational and translational self-motion

**Figure 4:**
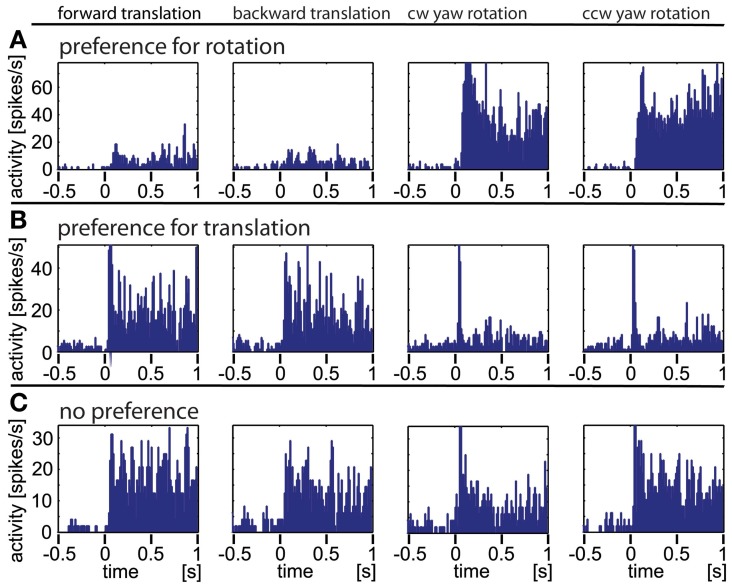
**Three typical examples of neurons responding to artificial optic flow stimuli.** The data were averaged over at least 30 repetitions, the bin size is 12 ms. The rows **(A–C)** correspond to the three neurons, the columns correspond to different motion directions (forward and backward translation as well as clockwise and counter-clockwise yaw rotation, respectively). Row **(A)** shows a neuron predominantly responding to rotational stimuli. **(B)** shows a neuron predominantly responding to translational stimuli with only transitional onset peaks for rotational stimuli. **(C)** depicts a neuron that responds to all four stimuli.

In Figure 4 we show the responses of three different example neurons obtained with the artificial optic flow stimuli. Neuron A responds strongly to rotational stimuli with both, a phasic and a tonic component and only weakly to translational movements. Neuron B reacts to translational stimuli with a small phasic and a strong tonic response; it reacts to rotational stimuli with only a sharp phasic response at the onset of movement. While these two neurons appear to react either to rotational or to translational stimuli, and obviously do not differentiate clearly between the directions of the stimulus movement (forward/backward, clockwise/counter-clockwise), the third neuron C shows similar responses to rotational and translational stimuli. Taken together, three response types were found during the presentation of artificial optic flow stimuli: a phasic response to motion onset, a tonic response to on-going motion and a combination of both. A single neuron could respond to each motion stimulus type differently, and by this in some cases revealed a preference for either rotational or translational stimuli.

**Figure 5:**
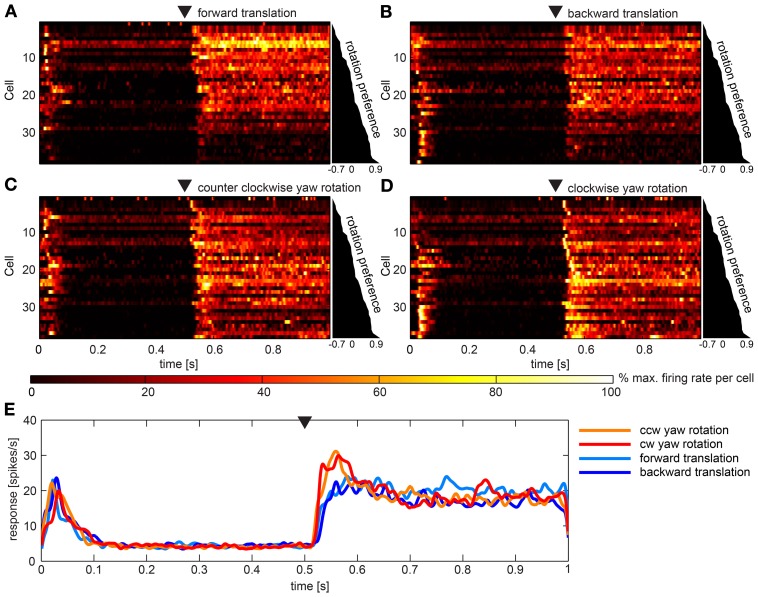
**Summary of the population data gathered using artificial optic flow stimuli. (A–D)** show response of 36 cells (y-axes) over time that responded to at least one of the four artificial optic flow stimuli with a firing rate 2 standard deviations above baseline. Cells are ranked by a preference score with top cells preferring the translational stimuli and bottom cells preferring rotational stimuli (diagram to the right of each panel indicates scores). For each cell the firing rate was normalized to its highest value within responses to all four stimuli and smoothed with a Gaussian filter (26 ms window; sigma = 3 ms). Colors reflect percent of maximum activation of the single cell as indicated in the color bar. Black triangle above the plot indicates motion onset. The corresponding gaze shift is **(A)** forward translation, **(B)** backward translation, **(C)** counter-clockwise (ccw) yaw rotation and **(D)** clockwise (cw) yaw rotation. **(E)** shows responses to the four stimuli averaged across the same neurons as above (not normalized), smoothed with a Gaussian filter (26 ms window; sigma = 3 ms). Black triangle indicates motion onset.

Figures 5A–D gives overviews of responses to the four artificial optic flow stimuli. Overall, 34 neurons (45%) of our sample responded to these optic flow stimuli. Each row shows the activation of one of these neurons color coded and normalized to the maximum activation of the same neuron across all stimuli. The cells are scored and sorted by preference for rotational stimuli. Scores were quite evenly distributed between −0.7 and 0.9 indicating a continuum of stimulus preference rather than an inherent separation of neurons preferring rotation or translation stimuli. Accordingly, during translational stimulation 13 neurons fired at more than twice the rate as during rotational stimuli (score < −0.33), while nine neurons responded preferentially to rotational stimuli (score > 0.33) and twelve neurons scored in between. In Figure 5E the average response to the four stimuli across all neurons is shown. It is apparent that tonic responses as averaged over the population did not differ, while rotational stimuli elicited phasic responses to motion onset. Turning on the stimulus (switch from one still image to another still image) in the beginning of the stimulus presentation elicited a short burst response in most cells. Nevertheless, a principle component analysis did not reveal reasonably small numbers of principle components that would further explain the variance between the responses.

The response to different artificial optic flow stimuli did not correlate with recording depth. Neurons responsive to whole field motion were found throughout the nucleus, but the different response types were unordered. Visually responsive neurons were found at the ventral border of nucleus rotundus (deeper than 5300 μm), but these cells did not respond to the stimuli used and were therefore disregarded. We do not have sufficient resolution to determine topography in the horizontal plane.

### Response to looming stimuli

**Figure 6:**
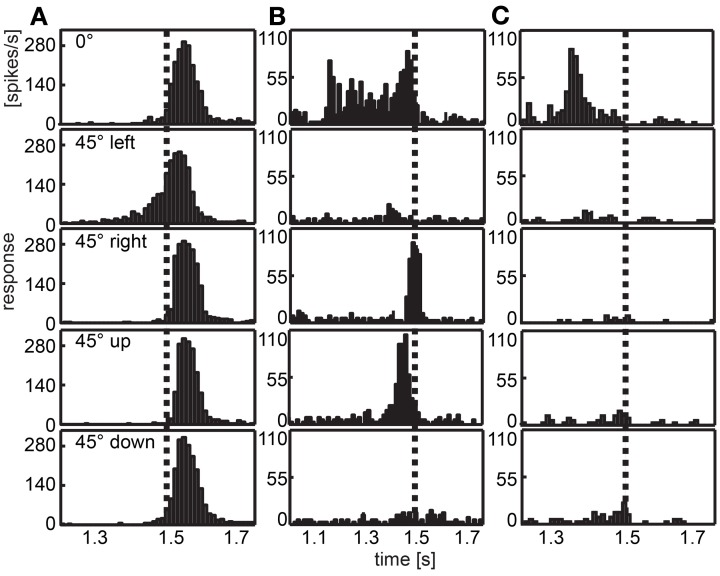
**Examples of response to looming objects.** Three examples of neurons are given that respond to a bright looming object in front of a dark background approaching from different directions. Histograms depict the response averaged over 30 repeats of each stimulus. The position of the object is indicated row-wise (frontal, at the center of the visual field; front left, 45° left of the center; front right, 45° right to the center; front up, 45° above the center; front down, 45° below the center). Vertical dotted lines indicate the end of the expansion stimulus. **(A)** Most neurons show a response peak shortly after the stimulus ended, when the panoramic display is completely lit. In this case, the response to the object positioned left to the center shows an earlier response. **(B,C)** are examples for neurons differentiating between the position of the object showing early response, no response or a response near to the end of the stimulus for different directions. Note different scalings of x- and y-axes.

Figure 6 illustrates the responses of three different cells chosen as examples. The neuron depicted in column A of Figure 6 responded to all expansion stimuli with a burst of action potentials shortly after the stimulus ended (stimulus end indicated by the vertical broken line). When the stimulus was positioned 45° to the left of the visual center (second panel), we found an exponential increment of spike activity. In Figure 6B a neuron is shown that responded to three of the stimuli (frontal, front right, and front up). When the expanding disc was placed in the center of the visual field (first panel), the spike rate was quite high relatively early and remained high until the stimulus ended. Two other stimuli elicited only brief bursts at stimulus offset (disc 45° to the right, third panel) or just before (disc above the center, fourth panel). The neuron depicted in Figure 6C responded only when the disc was positioned in the center of the visual field. Then the burst of spikes occurred earlier compared with most other responses.

**Figure 7:**
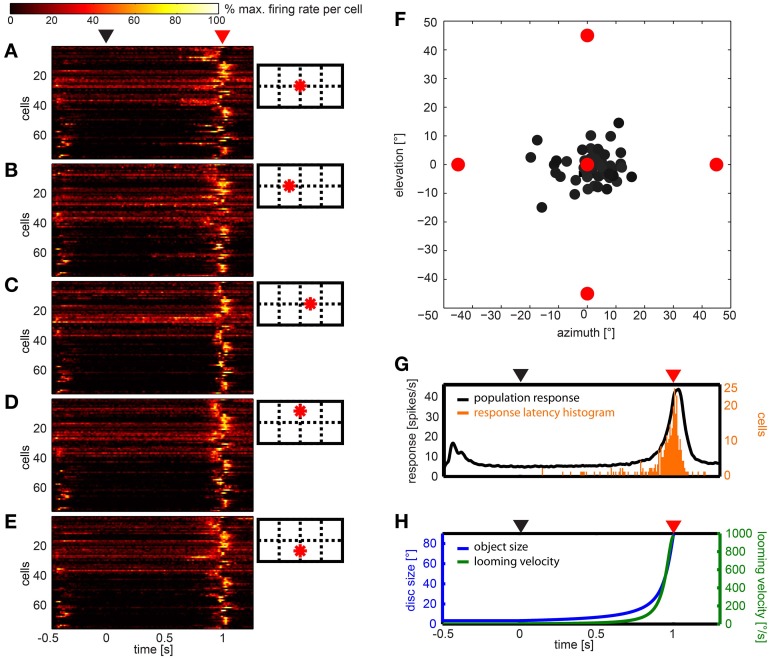
**Summary of the population data gathered using looming stimuli in different positions in the visual field. (A–E)** show response of 76 cells (y-axes) over time to each of the stimuli, respectively. For each cell and stimulus the firing rate was normalized to its highest value and smoothed with a Gaussian filter (26 ms window; sigma = 3 ms). Colors reflect percent of maximum activation of the single cell as indicated in the color bar above. Schemata to the right indicate origins of looming in the visual field. **(F)** Gray dots indicate the mean vector of the response to the looming discs per cell. Object origins are indicated by red filled circles. **(G)** Black line: population response across all stimuli (not normalized); smoothed with a Gaussian filter (26 ms window; sigma = 3 ms). Orange bars: histogram of onset of response during the looming stimulus. **(H)** Object size (°, blue curve) and looming velocity (°/s, green curve) during the stimulus for reference. **(A,G,H)** Black triangle above indicates looming onset, red triangle indicates collision time point.

Figures 7A–E show the response of all neurons to five different looming stimuli, respectively, normalized to each stimulus response. Twelve neurons (15.8%) did not respond to any of the five expansion stimuli. Eleven (14.5%) responded to the expansion stimulus at one of the five stimulus positions, four neurons (5.2%) responded to stimuli at two positions, four neurons (5.2%) responded to stimuli at three positions, ten neurons (13.2%) at four positions and the rest (35, 46%) at all five positions.

We calculated the mean response to the looming stimuli by summation of vectors describing each stimulus and the according response (Figure 7F). All vectors appear close to the center, a clear differentiation of cells into groups was not found. Plotting the 2d-projected angle of the mean response against rotational preference estimated from artificial optic flow stimuli did not reveal a correlation (not shown).

The histogram in Figure 7G (orange bars) shows that most response onsets occurred within the last 100 ms of the stimulus presentation (duration: 1 s) or up to 100 ms after virtual collision. The black line in Figure 7G indicates the firing rate averaged over the population data. As can be seen in Figure 7H, within this 200 ms time window, the retinal growth rate of the expanding disc exceeded 265°/s and its size reached and exceeded a diameter of up to 90° (by the end of the stimulus it fills the whole panoramic display). The response usually consisted of a single burst. There was no tonic activation found during the continued illumination (500 ms) at the end of the stimulus. To investigate whether the response latency simply signaled the time when the stimulus entered the receptive field, we sorted the data according to the distance between the stimulus origin and the center of the receptive field, where applicable (not shown). This did not reveal a correlation between position of the receptive field and response latency.

### Response to naturalistic optic flow stimuli

Seventy-four per cent (56 out of 76) of the neurons responded to naturalistic optic flow stimuli. The response patterns varied strongly between individual neurons with the exception that most of the neurons responded phasically to stimulus onset. We further did not find excitation or suppression of cell firing by fast head yaw rotations in any cells. Apart from this, the finding that cell responses were rather unique was strengthened by the attempt of hierarchical clustering on the complete data set. No clusters of multiple cells were clearly separable. Instead, repeated sorting did not reveal individual responses or cells that would end up as neighbors reliably. Only sporadic pairs of cells or responses were found that did not reveal obvious defining properties. We then looked at single cells and found several examples of cells differentiating between naturalistic stimuli that could further be explained by local disturbances in the optic flow.

#### The naturalistic stimuli revealed a high variance between cells

**Figure 8:**
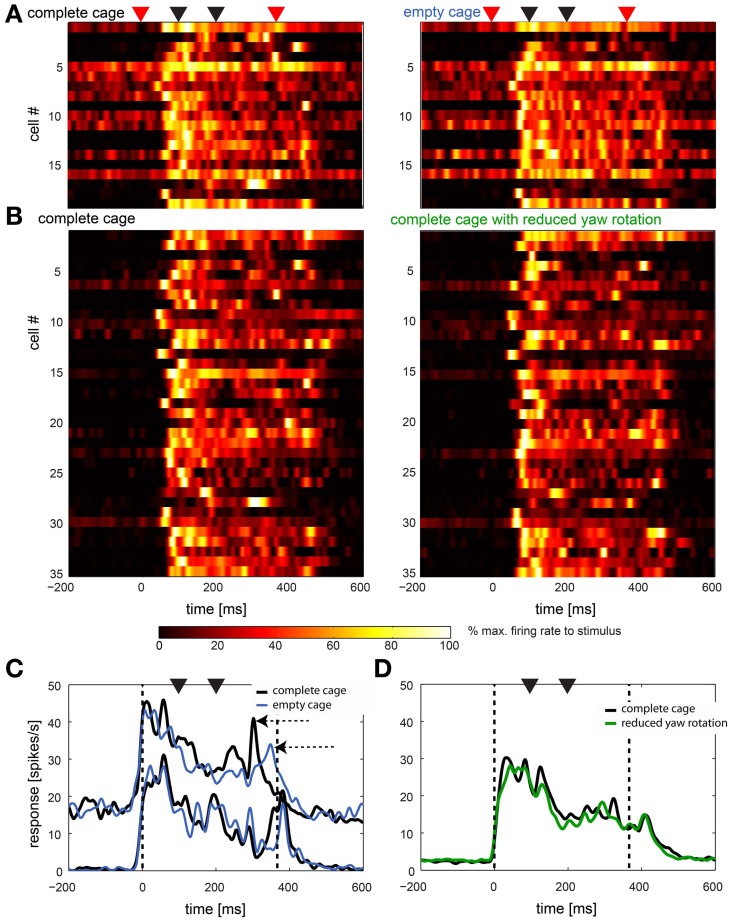
**Naturalistic optic flow stimuli: summary of response to complete cage stimulus in comparison to empty cage and reduced head yaw rotations. (A,B)** Left panels show the single cell response to the complete stimulus of all cells that were also stimulated with the empty cage stimulus (*n* = 19) and the stimulus with reduced head yaw rotations (*n* = 35), respectively (right panels). Each row represents a single cell. Cells in left and right panels are in the same order. Red triangles on top indicate beginning and end of the stimulus, black triangles indicate fast gaze shifts. Colors indicate firing rate normalized to maximum firing within each row. **(C,D)** show averaged population response in spikes/s over time for data shown in **(A,B)**, respectively (not normalized). Data were smoothed with a Gaussian filter (26 ms window; sigma = 3 ms). Vertical broken lines indicate beginning and end of the stimulus, black triangles indicate fast gaze shifts. Black lines indicates the response to the complete cage, blue lines in **(C)** indicate the response to the empty cage stimulus and the green line in **(D)** indicates response to the stimulus with reduced head yaw rotations between fast gaze shifts. Data in **(C)** is separated into two subpopulations based on baseline firing rate.

This high variability between cells can be seen in Figure 8 in which we present data from 44 cells in two comparisons. In **8A** we show the response of all cells (*n* = 19) that were stimulated with the complete cage stimulus (left panel) as well as the empty cage stimulus (right panel). In Figure 8B cells (*n* = 35) are shown that were presented the complete cage stimulus (left panel) and the one with reduced head yaw rotations (right panel). Note that some neurons were presented all three stimuli and therefore occur in A and B. Start and end of the stimulus are marked with red triangles while black triangles mark the occurrence of fast head yaw rotations.

Nevertheless, in Figures 8C,D we show responses averaged across the same sample shown in A and B. In Figure 8C we separated the cells in two subpopulations by their baseline activity. It appears that the averaged response of the sample with overall slower firing rate (*n* = 9) does not distinguish between stimuli while for the faster spiking neurons (*n* = 10) the averaged responses to the two stimuli differ (horizontal arrows). Figure 8D shows the response of the cells stimulated with the complete cage stimulus (black line) and the stimulus with reduced head yaw rotations (green line). Here, no difference in the response to the two stimuli is obvious. In general responses to the different naturalistic stimuli within the same cells appear to be more similar than responses of different cells to the same stimulus.

#### The response to artificial stimuli did not predict the response to naturalistic stimuli

We evaluated whether neurons that responded similarly to the artificial stimuli would also respond similarly to naturalistic stimuli. A single quantitative measure for the response to naturalistic stimuli cannot be determined. We therefore, sorted the naturalistic responses according to quantitative measures for the response to the artificial stimuli. These were the response latency to the artificial self-motion stimuli and the preference score for rotation and translation. We did not see patterns emerge when the data were plotted in the same fashion as in Figures 8A,B.

#### As the results indicated high variance between single cells we attempted to understand the responses of the individual neurons

**Figure 9:**
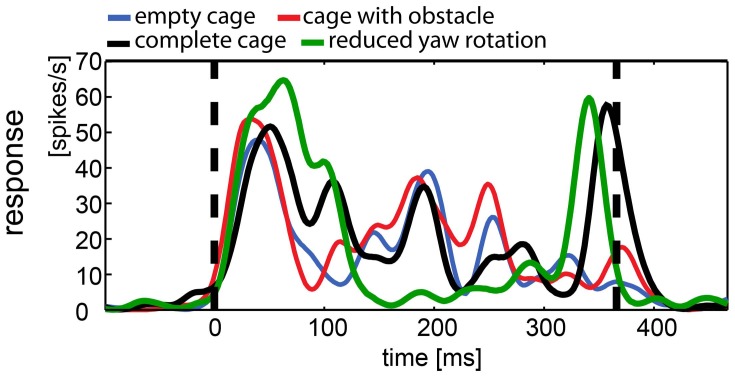
**Example of responses to naturalistic stimuli within a single neuron.** This neuron was presented all four naturalistic stimuli. The response to each stimulus was averaged over 35 repetitions and filtered by convolution with a one-dimensional Gaussian (sigma = 5 ms, window = 22 ms). Vertical broken lines indicate stimulus start and end. All stimuli elicited motion and onset response. During the stimulus with reduced head rotations (green line) the firing rate of the cell is less modulated than during the other stimuli with the exception of a salient peak in firing rate, coinciding with the approach toward the exit window. The “complete” stimulus serves as a control since it includes the same three-dimensional composition of the environment (black line; cage includes obstacle and exit windows). The response shows more modulation over the course of the stimulus but shares the peak response to the exit window. The other two stimuli did not include the exit window and lack the response to the window (blue, empty cage; red, cage with obstacle), but they share the modulation over time with the complete cage stimulus, regardless of the presence of the obstacle.

In contrast to averaged population responses, some single cells showed clear differences in the response to certain stimuli. We describe examples of single cell responses shown in Figure 9, which were also included in Figure 8. The response was modulated over the course of the stimulus. Figure 9 shows the response of a neuron to all versions of the naturalistic stimulus sequence. Apparently, the different stimuli caused different responses with a different time course. Motion onset elicited a strong response by all four naturalistic stimuli. In the empty cage condition (blue line) the response is modulated over the course of the stimulus. Adding the obstacle (red line) does not change the response in an obvious way. However, introduction of the exit window (complete cage, black line) adds a significant peak in response at the end of the stimulus, when the bird approaches the exit window. Interestingly, the response to the same stimulus without slow horizontal head motion (green line) lacks the modulation over the course of the stimulus. However, the exit window elicits the same peak in firing rate at the end of the stimulus.

#### None of the cells responded to optic flow induced by fast gaze shifts

**Figure 10:**
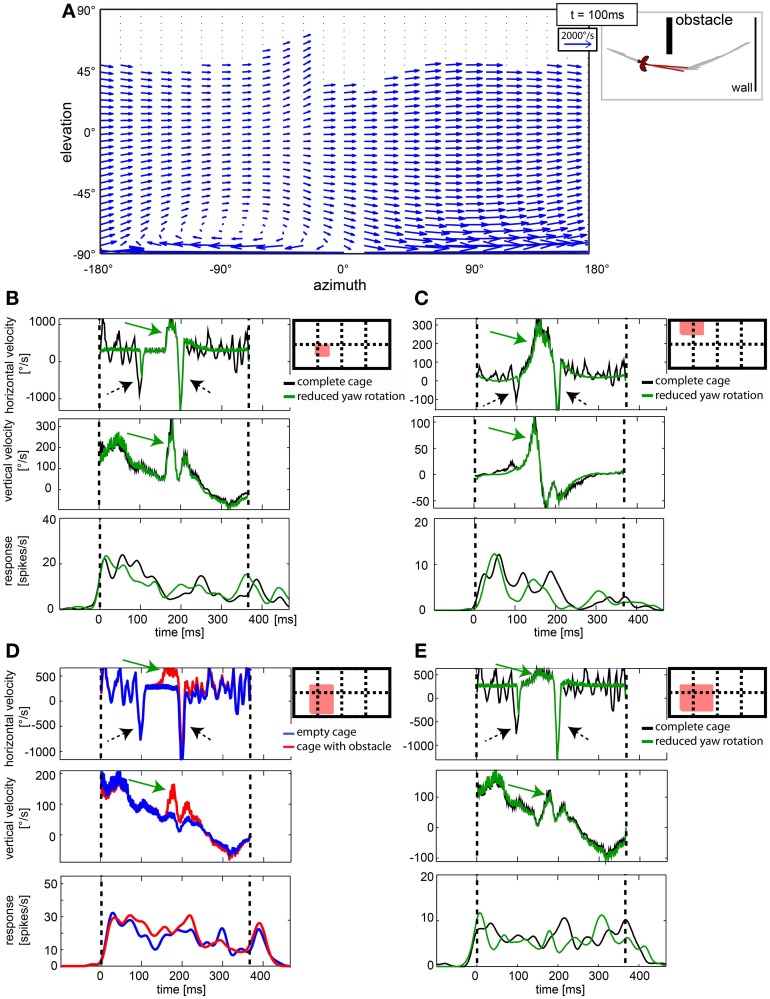
**Cells in nucleus rotundus did not respond to optic flow induced by fast gaze shifts. (A)** shows a vector plot of optic flow during a gaze shift to the left in cylindrical coordinates. The blue vectors indicate direction and velocity of local image displacements between two frames. The on-going gaze shift introduces fast image displacements wherever texture is visible in the scene. Y axis gives position in degrees of the elevation, X axis the same for azimuth. The schematic depicts position relative to objects in the arena and head orientation of the bird at the same moment (100 ms into the stimulus). **(B–E)** show in the right diagrams as a red filled rectangle the location of the receptive field of the analyzed neuron within the visual field [180° elevation, 360° azimuth; dotted lines are 90° apart]. The three plots show horizontal image velocity, vertical image velocity, and neural response, respectively. Velocities are averages of local image velocities at 5° distant sample points within the receptive field. Neural responses are averaged over 30 trials and smoothed by convolution with a one-dimensional Gaussian filter (sigma = 5 ms, window = 22 ms). Vertical lines indicate start and end of the stimulus. In the velocity plots, green arrows indicate object related changes in image velocity, whereas black arrows indicate changes induced by fast gaze shifts. In **(B,C,E)** the black curve corresponds to the complete stimulus that includes residual head rotations whereas the green curves depict the response to the reduced head rotation stimulus. In **(D)** the blue curves correspond to the empty cage stimulus whereas the red curves correspond to the cage with obstacle.

Gaze shifts caused significant shifts in the optic flow. However, neither the population data (Figure 7), nor the single cell analysis revealed cells that responded to fast gaze shifts. Figure 10A depicts the optic flow within the visual field during a saccadic gaze shift (at time 100 ms of the stimulus sequence). It is a vector plot depicting image motion within a hypothetical visual field, covering 360° azimuth and 180° elevation. A vector indicates the direction and the velocity of local image displacement (compare to the scale in the upper right corner). The fast gaze shifts caused strong horizontal image motion across the entire visual field with the exception of some dorsal parts in the visual field showing no image velocities. These parts were not covered with a texture that would allow motion detection (e.g., the open roof which is represented at the top of the stimulus field). Figures 10B–E show the averaged horizontal and vertical image velocities within the receptive field of a neuron and its response. The red square in the schemes of the visual field depicts the estimated position and size of the receptive field for each neuron. The cells in Figures 10B,C,E were presented the complete cage (black line) and the stimulus with reduced yaw rotations (green line) while the cell in Figure 10D was presented the empty cage (blue line) and the cage with obstacle (red line). The two saccadic gaze shifts are reflected in negative peaks of horizontal velocity (Figures 10B–E, stippled arrows). In many cases the velocity exceeded 1000°/s. However, none of the 76 neurons in our sample showed a response correlated with the fast horizontal gaze shifts (Figures 10B–E).

#### A subpopulation of cells responded to the disturbance of the optic flow by static objects

In Figures 10B–D, a passing object caused an additional (third) velocity maximum at about 170 ms which has a horizontal and a vertical component (green arrows). Whether this velocity peak causes a neuronal response is not clear from the graphs shown here. We therefore, investigated the response to the obstacles by comparing them to the responses to stimulus movies without the obstacle.

**Figure 11:**
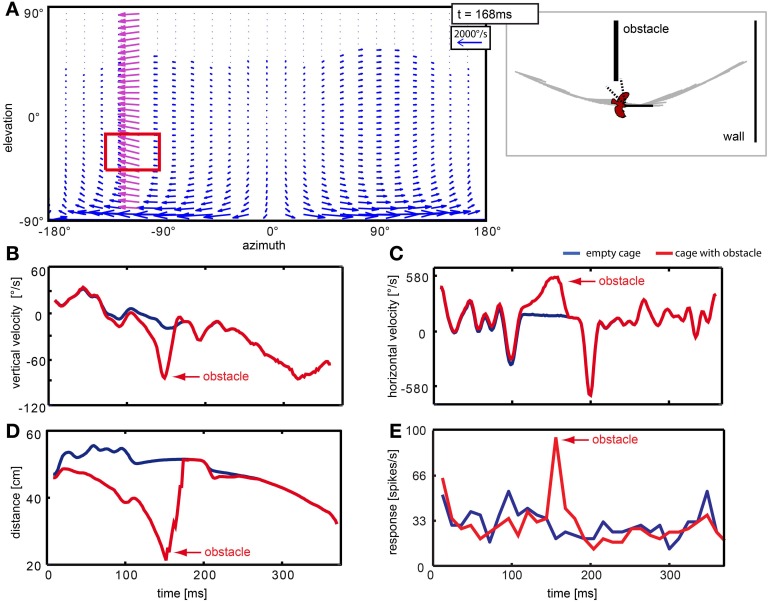
**Neuron responding to the obstacle in the left hemisphere of the visual field.** Panel **(A)** shows a vector plot of optic flow 168 ms after stimulus begin, when the bird is passing the object. The blue and purple vectors indicate direction and velocity of local image displacement between two frames. When the bird passed the obstacle in close proximity, the obstacle caused a local perturbance in the left hemisphere (purple vectors). The red rectangle indicates the receptive field of the neuron. The schematic at the right of the flow field depicts the position of the bird relative to objects in the arena and head orientation of the bird at the same moment. Dotted lines indicate the receptive field of the neuron. **(B–E)** Blue lines correspond to the empty cage stimulus; red lines correspond to the cage with obstacle. Red arrows point to changes that are related to the presence of the obstacle. **(B,C)** show local vertical and horizontal image velocities averaged over the receptive field. **(D)** shows the average distance between the bird and objects in the receptive field. **(E)** shows the response of this neuron to the two stimuli.

The obstacle distorted the optic flow pattern locally. Figure 11 depicts sample results of the velocities and corresponding neural responses with and without obstacle. Passing the obstacle generates a ‘wave’ of high velocity vectors moving across the neuron's receptive field (Figure 11A, red rectangle). The averaged vertical and horizontal velocity components over time within this region are plotted in Figures 11B,C, respectively. The blue line corresponds to the ‘empty cage’ where the objects (obstacle and windows) were eliminated. The red line corresponds to the stimulus that includes the obstacle (but not the windows). Prominent peaks (red arrows) occur in both velocity components for the obstacle condition. We also calculated the mean distance between the bird and objects (walls and obstacle; Figure 11D) within the receptive field for each frame and can conclude on this basis that high retinal velocities are caused by a small distance between bird and obstacle at the moment of passage (red arrow). The responses of the neuron (Figure 11E) to the two stimuli differ by a prominent response peak (red arrow) when the object was present in the receptive field.

**Figure 12:**
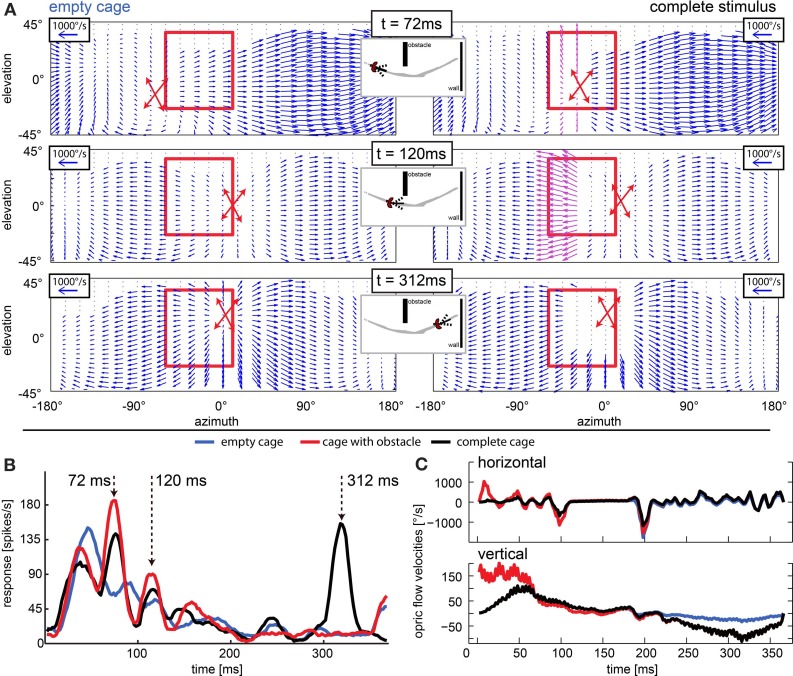
**Approach signaling neuron: response peaks occur when object position and point of expansion are collocated. (A)** Optic flow field at three moments of interest (rows; *t* = 72 ms, *t* = 120 ms, and *t* = 312 ms) for the empty cage (left column) and the stimulus including objects (right columns). The position of the point of expansion is marked by a red cross (estimated graphically from plot). The red rectangle indicates the receptive field of the neuron that responded as depicted in **(B)**. When the objects (obstacle and windows) are present, at 72 ms the point of expansion (center of red cross) is collocated with the obstacle (purple vectors), at 120 ms the local velocities generated by the obstacle are even stronger but the point of expansion is not collocated with the object. At 312 ms there are no velocities detectable close to the point of expansion but the edges of the window generate strong velocity vectors. **(B)** Response to three naturalistic test conditions each averaged over 30 repeats. Blue line corresponds to empty cage stimulus. Red line corresponds to the cage with obstacle and the black line corresponds to the complete stimulus. Arrows indicate peak responses of interest. Strongest responses are seen when the point of expansion is collocated with an object (*t* = 72 ms when the obstacle is present and *t* = 312 ms when the exit window is present). When the point of expansion is not collocated with the obstacle (*t* = 120 ms), the neuron responds only weakly although the obstacle produces high velocities in its receptive field. **(C)** Horizontal and vertical velocities averaged over the receptive field of the neuron [see **(A)**, red rectangle] over time. Blue line corresponds to empty cage stimulus; red line corresponds to the cage with obstacle, and the black line corresponds to the complete stimulus.

In cells that have receptive fields facing in flight direction, averaging velocities does not describe the overall flow well, because the point of expansion that emerges in heading direction consists of vectors that would cancel each other out. One such case is depicted in Figure 12. Figure 12A depicts the optic flow at three time points in the naturalistic stimuli. The left column shows the situation in the empty cage, the right column shows the same time point including obstacles and windows. The red crossed arrows indicate the point of expansion as it was estimated from the motion vectors in each figure by graphically following the velocity arrows back to their origin. When the bird is heading directly to an object (right column, *t* = 72 ms and *t* = 312 ms), the object is co-located with the point of expansion. The neuronal activity (Figure 12B) appears to correlate with an approach toward objects (obstacle and exit window) in the flight arena. The response to the empty cage (blue line) which does not include an obstacle or window shows no salient response peaks except a phasic response to motion onset. The response to the stimulus sequence that includes the obstacle but not a window is indicated by the red line. When the obstacle passed through the receptive field in the left visual field (Figure 12A, red rectangle) at 72 ms, it elicited a response. Then the bird performed a fast gaze shift to the left during which the obstacle moved back toward the location of the receptive field. Since the bird was still flying forward, the object entered the receptive field again at 120 ms, eliciting a smaller response. Finally, when the stimulus included the obstacle as well as the exit window (Figure 12B, black curve), we found the response peaks to correlate with the obstacle and a third one to correlate with the approach toward the exit window at 312 ms. We then compared the optic flow patterns between the peaks with the highest amplitudes (at 72 and 312 ms) to the one which was less prominent (120 ms). We found that the prominent responses occurred when the point of expansion was co-located with the object, which was the case at 72 and 312 ms, but not at 120 ms. In Figure 12C image velocities averaged over the receptive field of these neurons are plotted over time. They do not represent the presence of objects in the receptive field or a modulation that the neuronal response would be correlated with.

**Figure 13:**
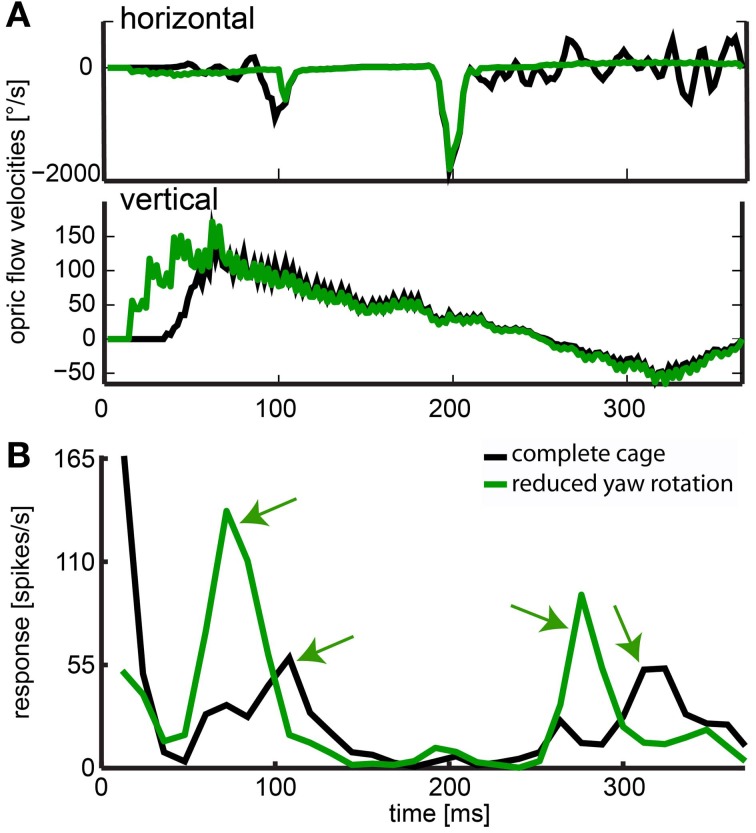
**Effect of removed horizontal head rotations. (A)** indicates horizontal and vertical velocities averaged across the receptive field for the complete cage stimulus (black line) and the stimulus with reduced head yaw rotation (green line). **(B)** The response of a neuron averaged over 30 repeats and filtered by convolution with a one-dimensional Gaussian filter (sigma = 5, window = 22 ms). The black line indicates response to the original flight trajectory in a cage including obstacle and windows; green line indicates response to the flight trajectory with removed horizontal head turns between saccadic gaze shifts in the same cage (reduced head rotations stimulus). Green arrows indicate object related response. These responses are much more salient when residual head rotations were eliminated (fast gaze shifts were included but not responded to by the neuron).

The stimulus with reduced head yaw rotations was designed to show whether increasing the separation of rotational and translational optic flow would enhance the saliency in the response to objects in the environment. The neuron depicted in Figure 13 showed significant responses to objects and it had a frontal receptive field. Like in the neuron just described, we found response peaks (black arrows) that could be correlated to the obstacle and exit window by viewing a video of vector maps of the optic flow in the receptive field of the neuron and the corresponding neural activity. With residual head rotations between saccadic gaze shifts (black curve), the spike rates in response to the objects were relatively low. Without residual head rotations (green curve), the response peaks reached much higher spiking rates and were more pronounced.

## Discussion

We are interested in depth perception from image motion in zebra finches during flight. As fast moving animals with small binocular fields, they are likely to rely on optic flow cues to perceive distances. We showed in a previous study, that zebra finches actively control their gaze in a way that would facilitate depth perception from optic flow (Eckmeier et al., 2008). We regard nucleus rotundus of the tectofugal visual system a candidate brain area for the processing of depth cues from optic flow. This expectation is based on previous studies that showed that nucleus rotundus and other areas in the tectofugal visual system code image motion differences between an object and a two-dimensional background (Frost et al., 1990; Wang et al., 1993; Wu et al., 2005; Xiao et al., 2006).

The methodological approach was borrowed from studies on insects and was focused on the presentation of artificial and naturalistic optic flow patterns that are consistent with realistic gaze shift and include depth information. The velocities in simulated self-motion were chosen to be in the range of real flight conditions. The optic flow was presented on a panoramic LED display to allow possible lateral modulation of neuronal responses within nucleus rotundus or maybe by other, whole field motion processing brain areas. This differs from other studies on birds which usually use moving gratings on a relatively small monitor or projector based stimulation that does not provide depth information.

As in pigeons (e.g., Wang et al., 1993; Laverghetta and Shimizu, 1999), at least a subset of nucleus rotundus neurons in zebra finches respond selectively to image motion. We observed only very low firing rates during presentation of a still image (preceding an optic flow stimulus), a dark display (not shown), or fully lit display (at the end of a looming stimulus), whereas sudden image changes elicit single bursts and continued motion elicited tonic firing. We could not localize a distinct subdivision of nucleus rotundus specialized in motion processing as was found in pigeons, for example by Wang et al. (1993). The lack of an anatomical clustering of motion selective neurons is in agreement with a lesion study of Laverghetta and Shimizu (1999). However, precisely locating recording sites in the relatively small nucleus rotundus in the zebra finch (1 mm diameter, Figure 2) is very difficult. We thus, do not challenge reports of compartmentalization of this nucleus in the zebra finch, or other birds (Nixdorf and Bischof, 1982; Wang et al., 1993; Laverghetta and Shimizu, 1999; Marin et al., 2003).

While not aiming for an in-depth analysis of neurons responding to looming stimuli, we used looming stimuli in order to identify cells that would potentially signal the approach of an object within the naturalistic stimulus. Again, we chose a velocity that would be in the range of natural flight velocity. Based on findings in pigeons looming cells would respond to a continuous looming stimulus within a range of expansion velocities. The response latencies in our population data indicate that the number of cells responding to a looming disc correlates with looming velocity (see Figure 6). The average firing rate across the population also followed the course of the looming stimulus. We first assumed that the cells responded when the stimulus entered their receptive fields. In this case, the response latency would be a function of the distance between the stimulus and the receptive field of the neuron. However, we did not find an obvious correlation of the distance between the stimulus and the receptive field and response onset. It is therefore, possible that the cells are tuned to a certain expansion velocity. Examination of the mean response vector also did not result in a classification based on the looming response. All mean vectors were close to the center which is probably due to the relative similarity between the looming stimuli. All stimuli originated in the frontal visual field.

We found excitatory responses to artificial optic flow stimuli. Most responses to the simplified visual flow fields were phasic-tonic, pure phasic responses were rare. A preference score revealed that the neuronal response in our dataset was evenly distributed across the whole range from strongly preferring rotational to strongly preferring translational stimuli (Figure 3). At this point we do not know how these differences in response preference emerge. Translational and rotational stimuli differed in the average motion velocity but also in the variance across motion vectors. Translational stimuli included many different angular motion vectors and rotational stimuli including only very similar motion vectors. The fact that rotational stimuli often elicited phasic response while translational stimuli did not elicit such an onset response might be due to the strong saliency of the whole visual field suddenly moving in the same direction in the rotational stimulus. We find it puzzling that neurons did not further differentiate between responses to stimuli of the same kind simulating movement in different directions. We presented many more translational stimuli for different directions (data not shown) which also did not reveal an impact of motion direction on neuronal response.

We think that the introduction of realistic motion patterns resulted in the finding of excitatory response to optic flow. Previous studies on motion processing in the nucleus rotundus only involved a two dimensional background and one object. When the object was moved in the same direction and with the same velocity as the background, no response was found (Frost et al., 1990). This seemed to implicate that whole field motion would suppress neuronal response to object movement. Moving a two dimensional background in front of the bird's eye, however, does not simulate either realistic translational gaze shift or rotational gaze shift. The stimuli used in these original studies further lacked depth information and did not cover a large portion of the visual field. We hypothesize that this response to realistic, complex motion patterns serves to modulate response to single objects for object to background differentiation and distance estimation. In this context, an object moving in accordance with a wall-like two dimensional background might simply not be interpreted as independent from the “wall” by the motion processing system, but rather as a part of its texture.

One goal for testing the artificial stimuli alongside with naturalistic stimuli was to attempt to predict the response to naturalistic stimuli from their response to the artificial ones. However, sorting the cells by response properties like latency, receptive field position, or preference for certain stimuli did not lead to obviously meaningful results. It is possible that combinations of these properties predict the naturalistic response, but our dataset is not large enough to test this. Another possibility is that the complexity of naturalistic stimuli leads to effects that cannot be predicted by a simple, linear model.

The majority of neurons were activated by naturalistic stimuli without actually responding to objects as they entered the neurons' receptive fields. We did, however, find a few cells that responded to the obstacle and/or the exit window. It is possible, that the naturalistic stimulus, although complex, only suited a small fraction of neurons. Regardless of the motion properties the neurons actually coded for, the distribution of preference scores for artificial optic flow stimuli indicates that neurons might not cluster in subpopulations that would signal only few, separate motion patterns. Instead, any motion pattern would be signaled, but only by a few neurons. This organization would reduce the probability of finding neurons responding to any one naturalistic stimulus. Finally, brain state probably plays a major role in the modulation of response properties in neurons. We found a subpopulation of fast firing neurons that appeared to show larger differences between naturalistic stimuli than a slow firing population (Figure 7C). Higher firing rates could be a result of lighter anesthesia, as we did not find fast and slow spiking neurons within the same animals. Studies on optokinetic response in pigeons further suggest that visual processing also changes between “flight” state and “resting” state (Maurice and Gioanni, 2004; Maurice et al., 2006). A broader variety of naturalistic stimuli but also recording from wake animals would probably increase the yield in following studies.

In the following we want to compare findings in insects with the results presented here. A behavior, similar to the saccadic gaze strategy we found in birds (Eckmeier et al., 2008) was already described for several insect species (Land, 1973; Hateren and Schilstra, 1999; Schilstra and Hateren, 1999; Boeddeker et al., 2010; Braun et al., 2010; Geurten et al., 2010; Kern et al., 2012). Electrophysiological studies on blowflies suggest that the separation of rotational and translational optic flow facilitates the processing of distances to objects in the environment (Kern et al., 2005; Karmeier et al., 2006; Egelhaaf et al., 2012; Liang et al., 2012). With regard to optic flow, response properties were described in flies that show similarities to those we found in the zebra finch, with the important exception that all the fly neurons are strictly directionally selective. For example, the so called H1 neuron responds to self-rotation (Krapp, 1999; van Hateren et al., 2005), VS and HS neurons encode ambiguous information on translation and rotation (Karmeier et al., 2006). The HSE neuron responds mainly to self-rotation if simplified rotational and translational stimuli are used. However, as Kern et al. (2005) found out, the same neuron, if stimulated by naturalistic motion sequences, exhibited the most intense and stimulus related responses not during the saccades of the fly (where the predominant motion is rotatory), but in the translational periods between saccades.

As found for the HSE neuron in flies, the response to simplified optic flow in nucleus rotundus of the zebra finch did not serve to predict the neuronal activity during the naturalistic optic flow stimulus. Strikingly, none of the neurons, including the rotation preferring ones, were activated during fast gaze shifts in the naturalistic stimulus, although there were very salient, high-velocity events in the optic flow (Figure 6). For mammals saccadic eye movements are known to suppress information probably using an efference copy (corollary discharge) of the eye movement (Von Holst, 1954; Bremmer et al., 2009). However, since our animals were anaesthetized, efference copies are unlikely to play a role. An alternative and probably more parsimonious explanation would be that the very high speeds of the saccades lie outside the range of velocities nucleus rotundus neurons are tuned to. We have shown previously in a behavioral study (Eckmeier and Bischof, 2008) that the fastest rotational movement a zebra finch follows with an optokinetical head rotation in a rotating drum with vertically oriented stripes is about 400°/s. The speed of the rotational flow induced by a saccade always exceeded 400°/s with peak velocities ranging up to ca. 2000°/s.

When stimulated with naturalistic scenes the HSE neurons of blowflies as well as some avian rotundal neurons react almost exclusively to environmental features during intersaccadic phases of straight gaze, i.e., if the optic flow is largely consistent with translational gaze shift. In both systems, it is the approach and the passage of obstacles which leads to strong changes of the activation pattern. We show here that some neurons increase firing when passing the obstacle; removal of the obstacle eliminates/cancels the corresponding response component (Figure 7). Likewise, the response of the HSE to a naturalistic optic flow stimulus is modulated by the distance between an object and a wall behind (Liang et al., 2008). This is a similar situation as in the studies which showed that velocity differences in background motion and object motion modulate response in nucleus rotundus and entopallium to moving objects (Frost et al., 1990; Xiao and Frost, 2009).

In our experiments on birds, neuronal responses caused by an approach toward an object were enhanced if the point of expansion of the translational flow field was co-located with the object within the receptive field of the recorded neuron (Figure 8). Although it may make sense that objects approaching from the front elicit stronger responses than those which are not in the direction of flight, we cannot provide an answer as yet to the question of how this difference is brought about. In the case of stimuli approaching the animal from different directions, the responses were very variable (Figure 4), and we did not find any special feature of the responses to objects approaching frontally.

In blowflies, a precise head—body coordination is essential for the visual system to separate the translational from the rotational optic flow in the fly (Kern et al., 2006). If this coordination is not good enough, the detection of objects on the basis of optic flow cues is strongly diminished. In flies, the eyes are fixed in the head, and thus a separation of the two optic flow components is a consequence of body—head interaction where the head movement adjusts the impreciseness of the body saccade. Indeed, Kern et al. (2006) were able to show that without the head correction the translational optic flow was contaminated too much by rotational components for extracting spatial information.

In zebra finches (Eckmeier et al., 2008) no body saccades were observed. The body is propagating in a smooth curve, and rotations introduced by this trajectory are compensated for by the head movement. The overall head direction during intersaccadic intervals, however, might not be fully constant in the zebra finch (Figure 1, Eckmeier et al., 2008). We found residual rotational fluctuations in the head movement which could be measurement errors or caused by imprecise head stabilization. In the latter case, eye movements could compensate for the stabilization error. In pigeons, eye movements during horizontal optokinetic reflex account for up to 20% of the total gaze shift (Gioanni, 1988). For zebra finches movements of the left and right eye are coupled in a way that could compensate imprecise head movements (Voss and Bischof, 2009). According to the idea of Kern et al. (2006), elimination of residual fluctuations would enhance the detectability of environmental features from the translational optic flow. To test this hypothesis in the zebra finch, we modified the naturalistic stimulus by keeping head orientation completely stable during intersaccadic intervals (red lines in Figure 1B). In some neurons this led to more salient responses to objects in the stimulus (Figure 9). A role of the eyes for the elimination of rotational residuals is thus plausible, although the evidence is as yet only indirect.

The comparison of zebra finches and blowflies demonstrate that similar optic flow processing mechanisms can be found in phylogenetically distant species that depend on optic flow for depth perception. The gaze control strategy in each species leads to a quite uncontaminated translational optic flow which is optimal for the extraction of spatial information during fast self-motion. Even at the neuronal level, evolution appears to have developed similar solutions, such as the lacking response to the rotational optic flow elicited by fast gaze shifts. While it is already possible to attribute self-motion and depth perception to a certain set of identified neurons in flies, the situation is still ambiguous in birds. Previously, it was assumed that only the accessory optic system processes optic flow. However, the accessory optic system seems to be only involved in self-motion processing (Simpson et al., 1988; Frost et al., 1990; Wylie, 2013). A very recently published study from Xiao and Frost (Xiao and Frost, 2013) implicates a role of depth perception also for self-motion processing. However, based on results from the same authors (Frost et al., 1990; Xiao and Frost, 2009), the tectofugal visual system still appears to be suited for the processing of the distance to objects. It is therefore, likely that both visual systems use motion cues to integrate depth information into the processing of self-motion as well as the signaling of objects.

We find that complex optic flow that is consistent with self-motion in a three dimensional environment elicits excitatory response in nucleus rotundus rather than only affect motion processing for single objects. Analysis of the response to a naturalistic optic flow scene was difficult and only few cells appeared to signal the objects in our set of stimuli. The sparse representation is probably due to the organization of motion representation in nucleus rotundus and effects of brain state on visual processing. However, the examples we describe indicate parallels between avian and insect optic flow processing. Our results lead us to many interesting questions about the processing of optic flow information, whether it is also apparent in other parts of the tectofugal visual system and by what mechanism optic flow information is integrated into object motion processing and object detection.

## Authorization for the use of experimental animals

The original research reported herein was performed under guidelines established by the German Welfare Law.

### Conflict of interest statement

The authors declare that the research was conducted in the absence of any commercial or financial relationships that could be construed as a potential conflict of interest.
